# Current News

**Published:** 1895-07

**Authors:** 


### Current News.

### AMERICAN DENTAL ASSOCIATION MEETING.

The Local Committee have made arrangements with the following hotels and houses for the session of the American Dental Association, commencing Tuesday, August 6, 1895. The "Coleman House," fronting the ocean, the largest hotel in Asbury Park, with rates $3.50 to $4.00 per day, and the use of the ballroom at stated periods of the day and evening for large committee meetings and sections. The "West End Hotel," a first-class hotel, opposite the "Coleman House," the rates $2.50 to $3.00 per day with the use of the ballroom at stated periods for committees and sections. The "Ocean Hotel," next door to the "West End," capacity nine hundred, first-class in every respect, rates based on one hundred or more Dental Convention guests at $2.50 to $3.50 per day with the use at stated periods of small parlors for committees. The "Hotel Brunswick," near the ocean front, first-class in every respect, from $3.00 per day up to $20.00 per week. This hotel has a series of small parlors on the main floor for committees and private receptions, the use of the same based on the number of Dental Convention guests at the hotel.

In the large hotels the use of the rooms for committees and sections is based on the contract of a certain number of guests, therefore the selection of a room ahead in some hotel is necessary. July 15 is the time most of the hotels would like to know. All hotels have electric lights, baths, and artesian-well water. In the small hotels the rates are: "The Grand Central," $1.50 to $2 50 per day and $14.00 to $16.00 per week. "The Ashland," $2.00 per day and $8.00 to $12.00 per week. "The Portland," $2.00 per day and $8.00 to $10.00 per week. "The Edgemere," $2.00 to $2 50 per day and $12.00 to $20.00 per week. "The Neptune," a nice quiet place, $1.75 to $2.00 per day and $9.00 to $20.00 per week. "The Albany," $2.00 per day and $8.00 to $15.00 per week. "The Clifton," $2.00 per day and $15.00 per week. "The Strand," $2.00 per day and $10.00 per week.

The committee will have an attendant at the Auditorium from July 27 to August 9, to give information in reference to hotels and other information to inquiring members. The trolley cars run direct from the depot to the Auditorium. A map of Asbury Park, the cuts of the hotels, and the Auditorium will appear in the New Jersey *Programme*, which will be mailed to every member of the American Dental Association.

Charles A. Meeker, *Chairman*,
C. W. F. Holbrook,
C. S. Stockton,
*Local Committee.*

---

### PENNSYLVANIA STATE DENTAL SOCIETY.

The next meeting of the Pennsylvania State Dental Society will be held at Eagle's Mere, Pennsylvania, July 9, 10, and 11, 1895.

Hotel accommodations have been secured at reduced rates.

An exceptionally good programme is at present promised, with papers and clinics by men of eminence in the profession.

Business of great importance will be presented and a new constitution is to be acted upon.

Give your profession the benefit of your judgment and make a sacrifice, if need be, to attend. Truly yours,

Howard E. Roberts,
*Corresponding Secretary.*

1321 Walnut Street, Philadelphia.

### AMERICAN DENTAL SOCIETY OF EUROPE.

The American Dental Society of Europe will hold its twentieth meeting at Boulogne-sur-Mer, France, August 5, 6, and 7, 1895. It is expected that there will be a very full attendance of the members, and that the meeting will be an interesting one. Any professional brethren who may be spending their holidays in Europe are cordially invited to make their plans so as to be present and take part with us at this meeting.

Programmes may be obtained of the President, Dr. Charles W. Jenkins, 1 Sonnenquai, Zürich, Switzerland, or of

Dr. J. H. Spaulding,
*Secretary.*

4 Rue de Rome, Paris.

---

### NATIONAL ASSOCIATION OF DENTAL FACULTIES.

The annual meeting of this body will be held at Asbury Park, New Jersey, on Saturday, August 3, at ten o'clock A.M. It is urged upon all colleges having membership to be promptly present at that hour, as much important business will be before the Association, and the time allotted is usually short for the work to be done.

The Executive Committee of the Association will meet on Friday previous, at ten o'clock A.M. at the same place. All business for that committee should, so far as possible, be in their hands before the meeting, in order that there be no delay.

J. Taft,
*Chairman Executive Committee.*
Louis Ottofy,
*Secretary.*

Masonic Temple, Chicago, Ill.

---

### TO ARRANGE FOR INTERSTATE MEETING, MISSOURI.

The committees appointed by the State Associations of Iowa, Nebraska, Colorado, Kansas, and Missouri to arrange for the Interstate Dental Meeting to be held at Excelsior Springs, May, 1896, will meet at Perth Springs, Missouri, July 10, 1895. A full attendance is desired.

J. P. Roots,
*Chairman General Committee.*
S. C. A. Rubey,
*Secretary.*

